# Social Media Fingerprints of Unemployment

**DOI:** 10.1371/journal.pone.0128692

**Published:** 2015-05-28

**Authors:** Alejandro Llorente, Manuel Garcia-Herranz, Manuel Cebrian, Esteban Moro

**Affiliations:** 1 Instituto de Ingeniería del Conocimiento, Universidad Autónoma de Madrid, Madrid 28049, Spain; 2 Departamento de Matemáticas & GISC, Universidad Carlos III de Madrid, Leganés 28911, Spain; 3 UNICEF Innovation Unit, New York, NY 10017, USA; 4 Department of Computer Science and Engineering, University of California at San Diego, La Jolla, CA 92093, USA; 5 National Information and Communications Technology Australia, Melbourne, Victoria 3003, Australia; University of Zaragoza, SPAIN

## Abstract

Recent widespread adoption of electronic and pervasive technologies has enabled the study of human behavior at an unprecedented level, uncovering universal patterns underlying human activity, mobility, and interpersonal communication. In the present work, we investigate whether deviations from these universal patterns may reveal information about the socio-economical status of geographical regions. We quantify the extent to which deviations in diurnal rhythm, mobility patterns, and communication styles across regions relate to their unemployment incidence. For this we examine a country-scale publicly articulated social media dataset, where we quantify individual behavioral features from over 19 million geo-located messages distributed among more than 340 different Spanish economic regions, inferred by computing communities of cohesive mobility fluxes. We find that regions exhibiting more diverse mobility fluxes, earlier diurnal rhythms, and more correct grammatical styles display lower unemployment rates. As a result, we provide a simple model able to produce accurate, easily interpretable reconstruction of regional unemployment incidence from their social-media digital fingerprints alone. Our results show that cost-effective economical indicators can be built based on publicly-available social media datasets.

## Introduction

Human behavior is closely intertwined with socioeconomical status, as many of our daily routines are driven by activities related to maintain, to improve, or afforded by such status [1–3]. From our movements around the city, to our daily schedules, to the communication with others, humans perform different actions along the day that reflect and impact their economical situation. The distribution of different individual behaviors across neighborhoods, municipalities, or cities impacts the economical development of those geographical areas, and in turn to that of the whole country [4–9]. Detecting patterns and quantifying relevant metrics to unveil the complex relationship between geography and collective behavior is thus of paramount importance for understanding the economical heart-beat of cities, and the structure of inter-city networks, and thus to economic planning, educational policy, urban planning, transportation design, and other large-scale societal problems [10–14].

Much knowledge about how mobility, social communication and education affect the economical development of cities has been being obtained through complex and costly surveys, with an update rate ranging from fortnights (unemployment) to decades (census) [15–17]. At the same time, the recent availability of vast and rich datasets of individual digital fingerprints has increased the scale and granularity at which we can measure these behavioral features, reduced the cost and update rate of these measurements, and provided new opportunities to combine them with more traditional socio-economical surveys [14, 18–22].

In this work we provide a proof of concept for the use of social media individual digital fingerprints to infer city-level behavioral measures, and then uncover their relationship with socioeconomic output. We present a comprehensive study of the different behavioral traces that can be extracted from social media: (i) technology adoption from (social media) user demographics, (ii) mobility patterns from geo-located messages, (iii) communication patterns from exchanged messages, and (iv) content analysis from the published messages. To this end, we use a country-scale publicly articulated social media dataset in Spain, where we infer behavioral patterns from almost 146 million geo-located messages. We match this dataset with the granular unemployment at the level of municipality, measured at the peak of the Spanish financial crisis (2012–2013). We consider unemployment to be the most important signal for the socioeconomic status of a region, since the effects of the crisis have had a very large impact in terms of unemployment in the country (around 9.2% in 2005, more than 26% in 2013).

Our extensive investigation of this large variety of traces in a large social media dataset allows us not only to build an accurate model of unemployment impact across geographical areas, but also to compare globally previously reported metrics in diverse works and datasets, as well as asses their relevance and uniqueness to understand economical development [14, 19–21, 23–28]. As we will show, technology adoption, mobility, diurnal activity, and communication style metrics carry a different weight in explaining unemployment in different geographical areas. Our goal is not to state causality between unemployment and the extracted metrics but to uncover the relationship emerging when we observe the economical metrics of cities and the social behavior at the same time.

## Results

### Social media dataset and functional partition of cities

Twitter is a microblogging online application where users can express their opinions, share content and receive information from other users in text messages of 140 characters long, commonly known as *tweets*. Users can interact with other users by mentioning them or retweeting (share someone’s tweet with your followers) their content. Some of these tweets contain information about the geographical location where the user was located when the tweet was published; we refer to them as geo-located tweets.

To perform our analysis, we consider 19.6 million geo-located Twitter messages (tweets), collected through the public API provided by Twitter from continental Spain, ranging from 29th November 2012 to 30th June 2013. Tweets were posted by (properly anonymized) 0.57 Million unique users and geo-positioned in 7683 different municipalities. We observed a large correlation (Pearson’s coefficient *ρ* = 0.951[0.949, 0.953]) between the number of geopositioned tweets per municipality and the municipality’s population. On average we find around 50 tweets per month and per 1000 persons in each municipality.

**Fig 1:**
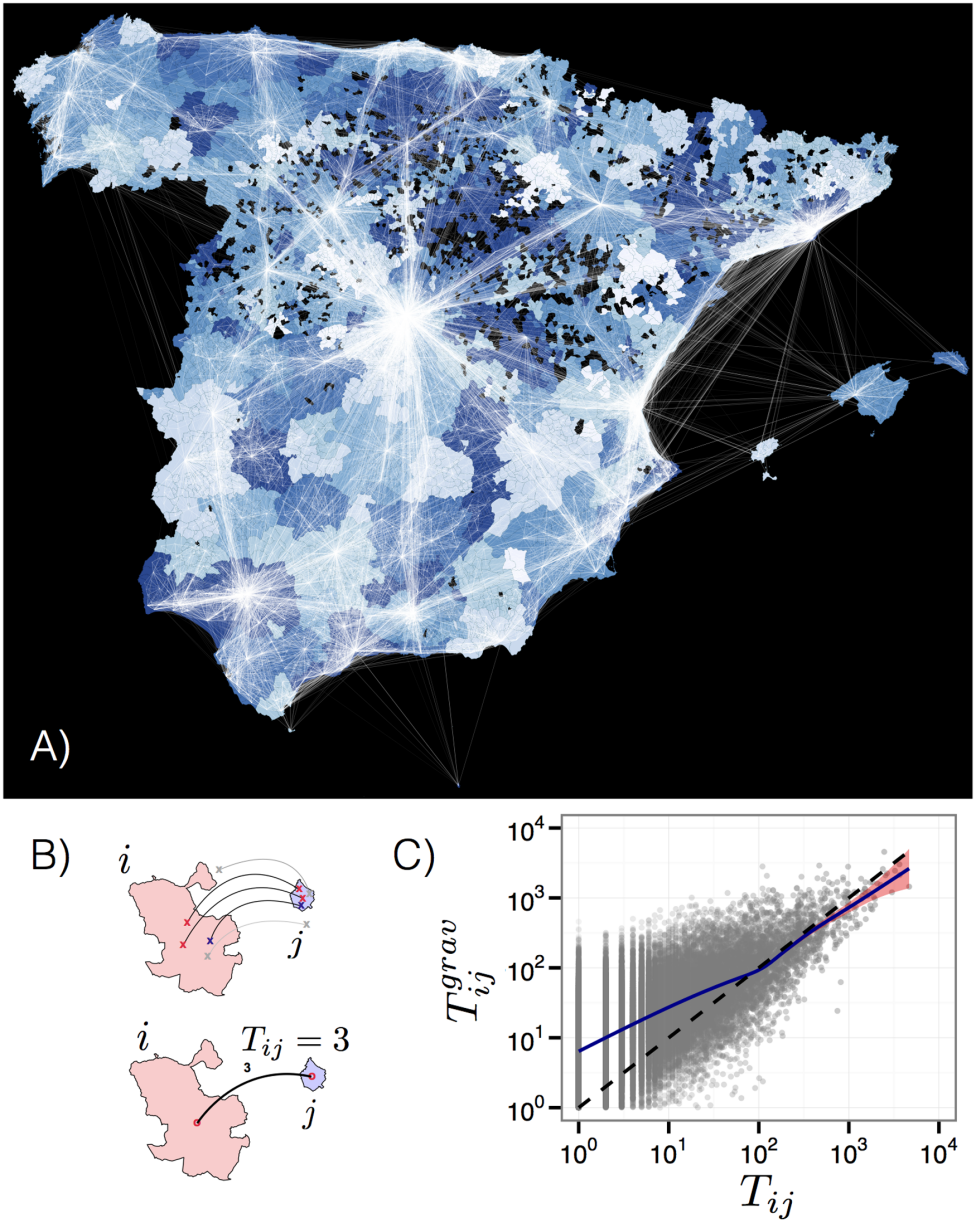
A) Map of the mobility fluxes *T*_*ij*_ between municipalities based on Twitter inferred trips (white). Infomap communities detected on the network *T*_*ij*_ are colored under the mobility fluxes (blue colors). B) Mobility fluxes *T*_*ij*_ between municipalities *i* and *j* are constructed by aggregating the number of trips between them. C) Correspondence between the observed fluxes *T*_*ij*_ and the fitted gravity model fluxes. Dashed line is the $T_{ij}=T_{ij}^{\text{grav}}$ while the (blue) solid line is an conditional average of $T_{ij}^{\text{grav}}$ for values of *T*_*ij*_. Maps were created using the maptools and sp packages in the R environment.

Despite this high level of social media activity within municipalities, we find their official administrative areas not suitable to study socio-economical activity: administrative boundaries between municipalities reflect political and historical decisions, while economical trade and activity often happens across those boundaries. The result is that municipalities in Spain are artificially diverse, ranging from a municipality with only 7 inhabitants to other with population 3.2 million. Although there exists natural aggregations of municipalities in provinces (regions) or statistical/metropolitan areas (NUTS areas), we have used our own procedure to detect economical areas. In particular, we have used user daily trips between pairs of municipalities as a measure of the economic relatedness between said municipalities. We say that there is a daily trip between municipality *i* and *j* if a user has tweeted in place *i* and *j* consecutively within the same day. In our dataset we find 1.9 million trips by 0.22 million users. With those trips we construct the daily mobility flux network *T*_*ij*_ between municipalities as the number of trips between place *i* and *j* (see Fig 1B). Remarkably, the statistical properties of trips and of the mobility matrix *T*_*ij*_ coincide with those of other mobility datasets (see Section B in S1 File): for example, trip distance *r* and elapsed time *δt* are power-law distributed with exponents *P*(*r*) ∼ *r* ^−1.67^ and *P*(*δt*) ∼ *δt* ^−0.67^, very similar to those found in the literature [9, 24]. And the mobility fluxes *T*_*ij*_ are well described by the Gravity Law (*R* ^2^ = 0.80) [29]

$$\begin{array}{c} T_{ij}≃T_{ij}^{\text{grav}}=\frac{P_{i}^{\alpha_{i}}P_{j}^{\alpha_{j}}}{d_{ij}^{\beta }} \end{array}$$ (1)

where *P*_*i*_ and *P*_*j*_ are the populations of municipalities *i* and *j* and *d*_*ij*_ is the distance between them. Similarly, the exponents in Eq (1) are very similar to those reported in other works *α*_*i*_ ≃ *α*_*j*_ = 0.48 and *β* ≃ 1.05 [24, 30]. These results suggest that detected mobility from geo-located tweets is a good proxy of human mobility within and between municipalities [31].

We use the network of daily fluxes between municipalities *T*_*ij*_ to detect the geographical communities of economical activity. To this end we employ standard partition techniques of the mobility network *T*_*ij*_ using graph community finding algorithms. This technique has been applied extensively, specially with mobile phone data, to unveil the effective maps of countries based on mobility and/or social interactions of people [32–34]. In our case, we have used the Infomap algorithm [35] and found 340 different communities within Spain. For further details about the comparison among different state-of-art community detection algorithms executed on the inter-city graph, see Section C in S1 File. The average number of municipalities per community is 21, and the largest community contains 142 municipalities. The communities detected have very interesting features (see Section C in S1 File): (i) they are cohesive geographically (see Fig 1), (ii) they are statistically robust against randomly removal of trips in our database (Table B in S1 File), and (iii) modularity of the partition is very high (0.76, see Table C in S1 File). Finally, (iv) the partition found has some overlap (77% of Normalized Mutual Information, NMI, see [36]) with coarser administrative boundaries like provinces (regions) (see Section C in S1 File for details). But interestingly, it shows a larger overlap (83% of NMI) with *comarcas* (counties), areas in Spain that reflect geographical and economical relations between municipalities. This result shows that the mobility detected from geo-located tweets and the communities obtained are a good description of economical areas. In the rest of the paper, we restrict our analysis to the geographical areas defined by the Infomap detected communities (see Fig 1). For statistical reasons, we discard communities which are not formed by at least 5 municipalities. Despite this sampling, 96% of the total country population is considered in our analysis. Our results in the rest of the paper also hold for municipalities, counties or provinces (though with lower statistical power) and communities detected by other community-finding algorithms (see Section I in S1 File).

### Social media behavioral fingerprints

The goal of this work is to quantify how and what behavioral features can be extracted from social media and then related back to the to the economical level of cities. To this end, we define four groups of measures that have been widely explored in other fields like economy or social sciences. These four types measures rely on the identification of the place where users live. Instead of using information in the user profile, we analyze the places where the user has tweeted and we set as *hometown* of the user the municipality where he/she has tweeted with the highest frequency, a method usually employed in mobile phone and social media [11, 24]. To this end we select those users with more than 5 geo-located tweets in our period and which have tweeted at least 40% of their tweets in a given municipality, which we will consider their hometown. After this filtering we end up with 0.32 million users and we can then define the twitter population *π*_*i*_ in area *i* as the number of users with their hometown within area *i*. We obtain a very high correlation between *π*_*i*_ and population of the cities *P*_*i*_ in the national census *ρ* = 0.977[0.976, 0.978] which provides an indirect validation of our approach with the present data. However not all demographic groups are equally represented in the our twitter database. As shown in Section D in S1 File, Twitter user demographics in Spain obtained from surveys [37] show that age groups above 44 years old are under-represented. Thus our results would mainly describe the socio-economical status of people below 44 years old. Employment analysis is then performed in different age groups: unemployment for people below 25 years old, between 25 and 44 years old and older than 44 years old. Finally, we have chosen the unemployment reported officially at the end of our observation time window (June 2013), but our results are not affected by the month selected, see Section G in S1 File.

**Fig 2:**
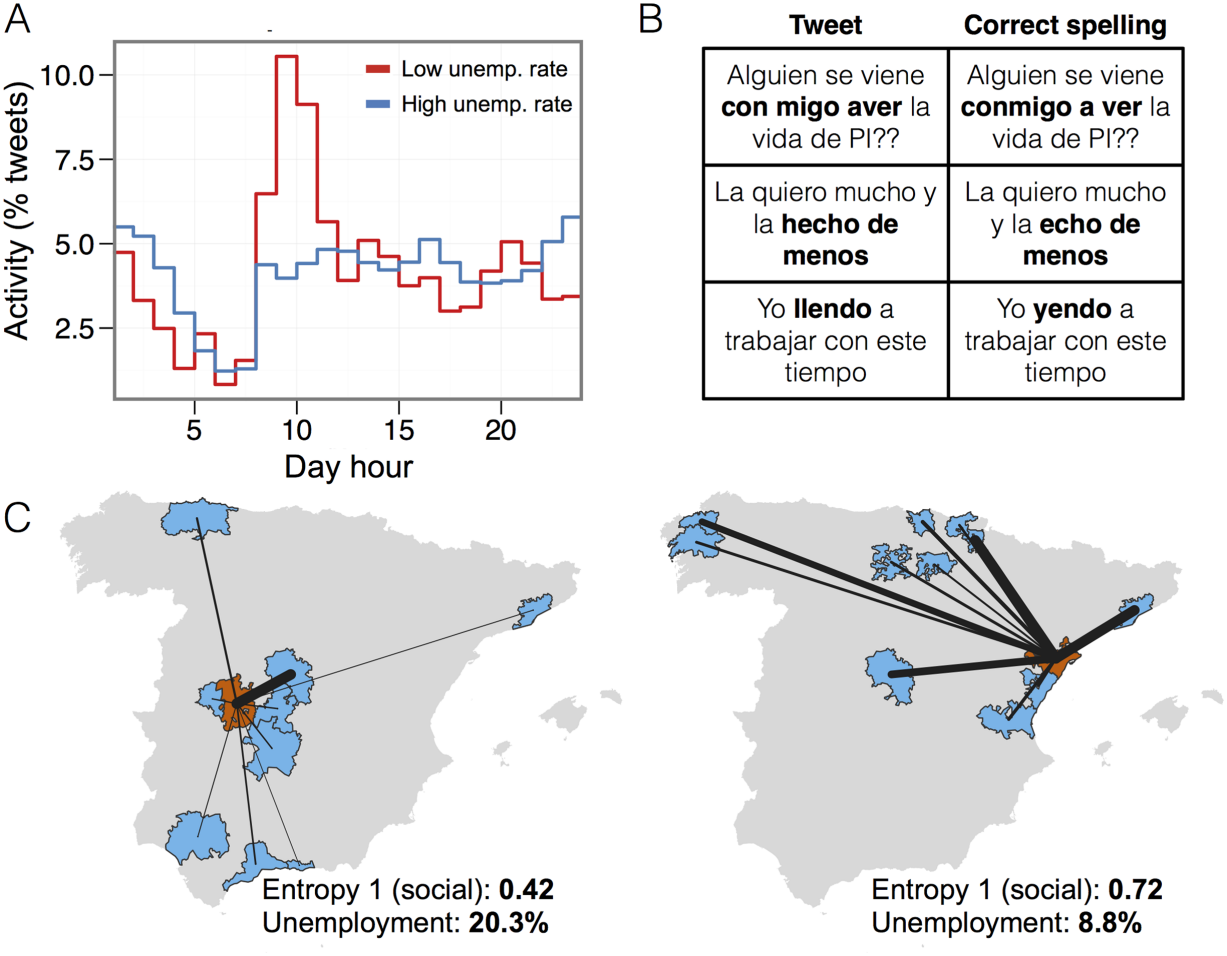
A) Correlation coefficient of all the extracted Twitter metrics grouped by technology adoption (black) geographical diversity (orange), social diversity (light blue), temporal activity (green) and content analysis (dark blue). Error bars correspond to 95% confidence intervals of the correlation coefficient. Gray area correspond the statistical significance thresholds. Panels B, C, D and E show the values of 4 selected variables in each geographical community against its percentage of unemployment. Size of the points is proportional to the population in each geographical community. Solid lines correspond to linear fits to the data.

**Fig 3:**
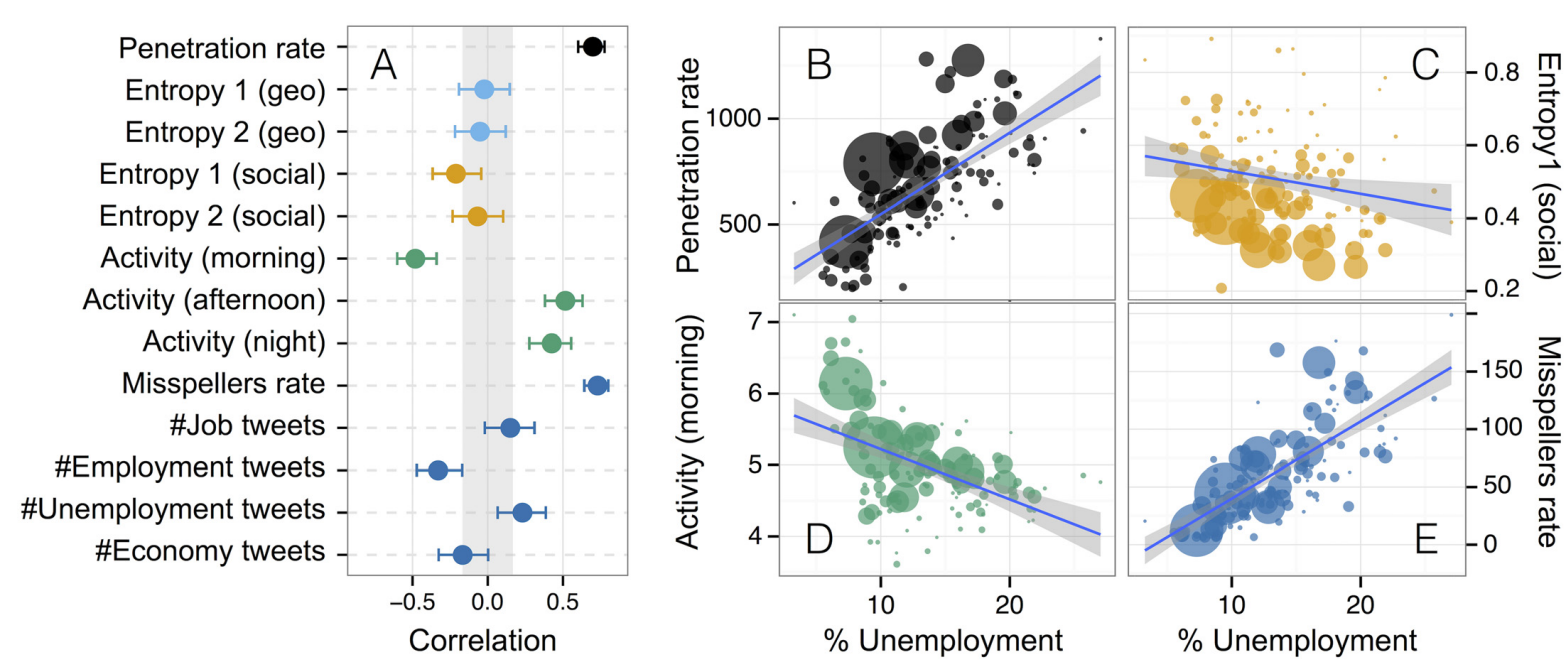
Examples of different behaviour in the observed variables and the unemployment. In A, we observe that two cities with different unemployment levels have different temporal activity patterns. Panel C show how communities (red) with distinct entropy levels of social communication with other communities (blue) may hold different unemployment intensity: left map shows a highly focused communication pattern (low entropy) while right map correspond to a community with a diverse communication pattern (high entropy). Finally, Panel B shows some examples of detected misspellings in our database using 618 incorrect expressions (see Section F in S1 File) such as “Con migo”, “Aver” or “llendo”.

For every considered region, we investigate the officially reported unemployment for different age groups and a number of metrics related to social media activity. Some of those metrics are already reported in the literature, but some others are introduced in this work. Specifically we consider:

- *Social media technology adoption*: we can use twitter penetration rate *τ*_*i*_ = *π*_*i*_/*P*_*i*_ in each area *i* as a proxy of technology adoption. Recent works have shown that indeed there is a correlation between country GDP and twitter penetration: specifically, it was found that a positive correlation between *τ*_*i*_ and GDP at the country level [24]. However, in our data we find the opposite correlation (see Fig 2), namely, that the larger the penetration rate the bigger the unemployment is, which suggest that the impact of technology adoption at country scale is different of what happens within an (industrialized) country where technology to access social media is commoditized.
- *Social media activity*: regions with very different economical situations should exhibit different patterns of activity during the day. Since working, leisure, family, shopping, etc. activities happen at different times of the day, we might observe different daily patterns in regions with different socio-economical status. For example, we hypothesize that communities with low levels of unemployment will tend to have higher activity levels at the beginning of a typical weekday. This is indeed what we find: Fig 3A shows the hourly fraction of tweets during workdays of two communities with very different rate of unemployment. As we can observe, both profiles are quite different and, in the case of low unemployment, we find a strong peak of activity between 8 and 11am (morning), and lower periods of activity during the afternoons and nights. We encode this finding in *ν*_mrng,*i*_, *ν*_aftn,*i*_, and *ν*_ngt,*i*_ the total fraction of tweets happening in geographical area *i* between 8am and 10am, 3pm and 5pm, and 12am and 3am respectively. Fig 2 shows a strong negative correlation between *ν*_mrng,*i*_ and the unemployment for the communities in our database and positive correlation with *ν*_aftn,*i*_, and *ν*_ngt,*i*_.
- *Social media content*: some works have observed a correlation between the frequency of words related to work conditions [21] or Google searches [22, 23] to unemployment or economical situation of countries. In our case we also find that there is a moderate positive correlation between the fraction of tweets *μ*_*i*_ mentioning *job* or *unemployment* terms and the observed unemployment, while the correlation is negative for the number mentions to *employment* or the *economy*. However, we have tried a different approach by measuring the relation between the way of writing and the educational level [38]. To this end, we build a list of 618 misspelled Spanish expressions and extract the tweets of the dataset containing at least one of these words (see Section F in S1 File for further details about how these expressions were collected). We only consider tweets in Spanish, detected with a N-grams based algorithm. Then, we only consider misspellings that cannot be justified as abbreviations. Finally, we compute for every region the proportion *ɛ*_*i*_ of *misspellers* among the Twitter population. If the fraction of misspellers per geographical area is a proxy for the educational level of that region, we expect a positive correlation between *ɛ*_*i*_ and unemployment. Indeed we find (see Fig 2) that there is a strong correlation between the fraction of misspellers and unemployment.
- *Social media interactions and geographical flow diversity*: following the ideas in [14] which correlated the economical development of an area with the diversity of communications with other areas, we consider all tweets mentioning another user and take them as a proxy for communication between users. Then we compute the number of communications *w*_*ij*_ between areas *i* and *j* as the number of mentions between users in those areas. To measure the diversity we use as in [14] the informational normalized entropy (Entropy 1) *S*_*u*,*i*_ = −∑_*j*_ *p*_*ij*_ log *p*_*ij*_/*S*_*r*,*i*_ where *p*_*ij*_ = *w*_*ij*_/∑_*j*_ *w*_*ij*_ and (Entropy 2) *S*_*r*,*i*_ = log *k*_*i*_ with *k*_*i*_ the number of different areas with which users in area *i* have interacted. As in [14], we find that areas with large unemployment have less diverse communication patterns than areas with low unemployment. This translates in a moderate negative correlation between *S*_*i*_ and the unemployment, see Fig 2. Similar ideas are applied to the flows of people between areas to investigate the diversity of the geographical flows through the entropy ${\tilde{S}}_{i}=-\sum_{j}{\tilde{p}}_{ij}\text{log}{\tilde{p}}_{ij}/{\tilde{S}}_{r,i}$, where ${\tilde{p}}_{ij}=T_{ij}/\sum_{j}T_{ij}$ and $S_{r,i}=\text{log}({\tilde{k}}_{i})$ with ${\tilde{k}}_{i}$ the number of different areas which has been visited by users that live in area *i*. Fig 2 shows that as in [19], correlation of these geographical entropies is low with economical development.

Normalization of variables is discussed in Section E in S1 File. We have also studied the correlation between the variables considered. As expected, variables in each group show moderate correlations between them. However the inspection of the correlation matrix and a Principal Component Analysis of the variables considered, show that there is information (as percentage of variance in the data) in each of the groups of variables, see Section E in S1 File. Because of these two facts we restrict our analysis to the variables within each group with the highest correlation with the unemployment, namely the penetration rate *τ*_*i*_, the social and mobility diversity variables *S*_*u*,*i*_ and ${\tilde{S}}_{u,i}$, the morning activity *ν*_mrng,*i*_, the fraction of misspellers *ɛ*_*i*_ and fraction of *employment*-related tweets *μ*_*emp*,*i*_.

### Explanatory power of social media in unemployment

The four previous groups of variables are fingerprints of human behavior reflected on the Twitter usage habits. As we observed in Fig 2, all of them exhibit statistically strong correlations with unemployment. The question we address in this section is whether those variables suffice to explain the observed unemployment (their explanatory power) and also determine the most important ones among themselves (which give more explanatory power than others). Note that we are not stating a causality arrow between the measures built in the previous section and the unemployment rate but only exploring whether they can be used as alternative indicators with a real translation in the economy.

**Fig 4:**
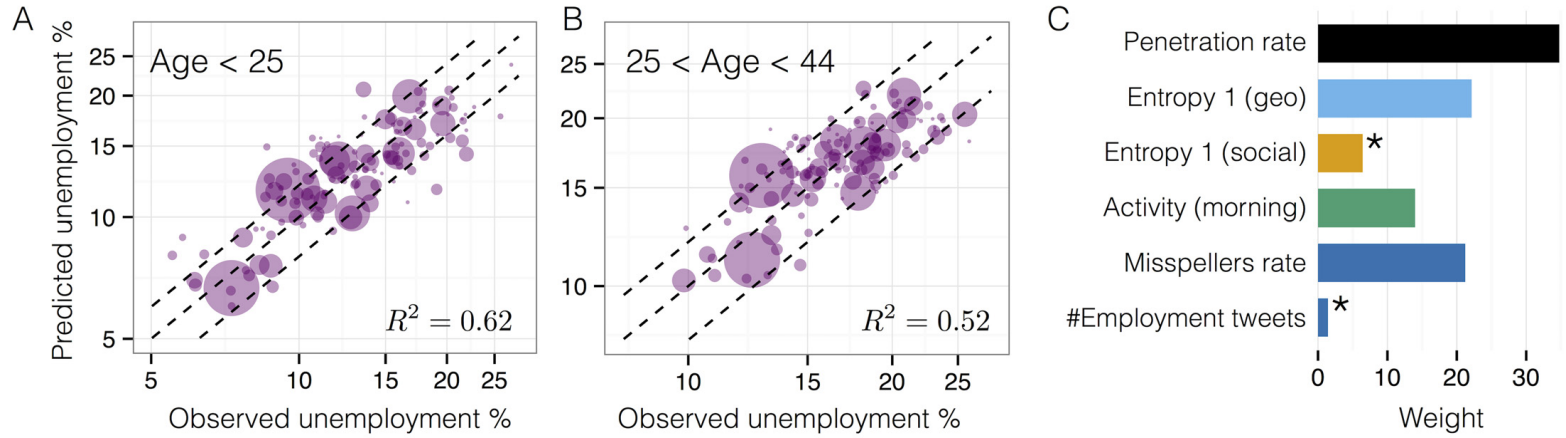
A) and B) Performance of the model, showing the predicted unemployment rate for ages below 25 versus the observed one, *R* ^2^ = 0.62 and with ages between 25 and 44. Dashed lines correspond to the equality line and ±20% error. C) Percentage of weight for each of the variables in the regression model using the relative weight of the absolute values of coefficients in the regression model (see Section J in S1 File). Variables marked with * are not statistical significant in the model.

Fig 4 shows the result of a simple linear regression model for the observed unemployment for ages below 25 years as a function of the variables which have more correlation with the unemployment. The model has a significant *R* ^2^ = 0.62 showing that there is a large explanatory power of the unemployment encoded in the behavioral variables extracted from Twitter. However, not all the variables weight equally in the model: specifically, the penetration rate, geographical diversity, morning activity and fraction of misspellers account for up to 92% of the explained variance, while social diversity and number of *employment* related tweets are not statistical significant (see Section J in S1 File for the methods used to determine the relative importance of the variables). It is interesting to note that while social diversity obtained by mobile phone communications was a key variable in the explanation of deprivation indexes in [14, 19], the communication diversity of twitter users seem to have a minor role in the explanation of heterogeneity of unemployment in Spain.

Similar explanatory power is found for other age groups: *R* ^2^ = 0.44 for all ages and *R* ^2^ = 0.52 for ages between 25 and 44 years. However, the model degrades for ages above 44 years (*R* ^2^ = 0.26) proving that our variables mainly described the behavior of the most represented age groups in Twitter, namely those below 44 years old. On the other hand, since our Twitter variables seem to describe better behavior of young people, we have investigated whether Twitter constructed variables have similar explanatory value (in terms of *R* ^2^) than simple census demographic variables for young people. As we can see in Section H in S1 File if we include the young population rate in our model we get a minor improvement *R* ^2^ = 0.65; on the other hand a model based only on young population rate gives *R* ^2^ = 0.24. This semi-partial analysis shows that Twitter variables do indeed posses a genuine explanatory power away from their simple demographic representation. Finally, our model have the largest explanatory power for detected communities, but large *R* ^2^ are also found for other detected communities (*R* ^2^ = 0.61) and other geographical areas like counties (*R* ^2^ = 0.54) and provinces (*R* ^2^ = 0.65), see Section I in S1 File.

## Discussion

This work serves as a proof of concept for how a wide range of behavioral features linked to socioeconomic behavior can be inferred from the digital traces that are left by publicly-available social media. In particular, we demonstrate that behavioral features related to unemployment can be recovered from the digital exhaust left by the microblogging network Twitter. First of all, Twitter geolocalized traces, together with off-the-shelve community detection algorithms, render an optimal partition of a country for economical activity, showing the remarkable power of social media to understand and unveil economical behavior at a country-scale. This insight is likely to apply to other administrative definitions in other countries, specially when considering large cities with an inherent dynamical nature and evolution of mobility fluxes, and cities composed of small satellite cities with arbitrary agglomerations or division among them (e.g. London, NYC, Singapore). This result is unsurprising: it should be natural to recompute city clusters/communities of activity based on their real time mobility, which may vary considerably faster than the update rates of mobility and travel surveys [32–34].

Our main result demonstrates that several key indicators, different penetration rates among regions, fingerprints of the temporal patterns of activity, content lexical correctness and geo-social connectivities among regions, can be extracted from social media, and then used to infer unemployment levels. These findings shed light in two directions: first, on how individuals’ extensive use of their social channels allow us to characterize cities based on their activity in a meaningful fashion and, secondly, on how this information can be used to build economic indicators that are directly related to the economy. Regarding the latter, our work is important for understanding how country-scale analysis of Social Media should consider the demographic but also the economical difference between users. As we have shown, users in areas with large unemployment have different mobility, different social interactions, and different daily activity than those in low unemployment areas. This intertwined relationship between user behavior and employment should be considered not only in economical analysis derived from social media, but also in other applications like marketing, communication, social mobilization, etc.

It is particularly remarkable that Twitter data can provide these accurate results. Twitter is, among the many currently popular social networking platforms, perhaps the noisiest, sparsest, more ‘sabotaged’ medium: very few users send out messages at a regular rate, most of the users do not have geolocated information, the social relationships (followers/followers) contains a lot of unused/unimportant links, it is plagued by spam-bots, and last but not least, we have no way to identify the motive/goal/functionality of mobility fluxes we are able to extract. These limitations are not particular to our sample, but general to the sample Twitter data being employed in the computational social science community. Despite all these caveats, we are able to show that even some simple filtering techniques together with basic statistical regressions yields predictive power about a variable as important as unemployment. Other social media platforms such as Facebook, Google+, Sina Weibo, Instagram, Orkut, or Flicker with more granular and consistent individual data are likely to provide similar or better results by themselves, or in combination. Further improvements can be obtained by the use of more sophisticated statistical machine learning techniques, some of them even tailored to the peculiarities of social media data. Our work serves to illustrate the tremendous potential of these new digital datasets to improve the understanding of society’s functioning at the finer scales of granularity.

The usefulness of our approach must be considered against the cost and update rate of performing detailed surveys of mobility, social structure, and economic performance. Our database is publicly articulated, which means that our analysis could be replicated easily in other countries, other time periods and with different scopes. Naturally, survey results provide more accurate results, but they also consume considerably higher financial and human resources, employing hundreds of people and taking months, even years to complete and be released—they are so costly that countries going through economic recession have considered discontinuing them, or altering their update rate in recent times. A particularly problematic aspect of these surveys is that they are “out-of-sync” i.e. census may be up to date, whereas those same individuals’ travel surveys may not be, and therefore drawing inferences between both may be particularly difficult. This is a particularly challenging problem that the immediateness of social media can help ameliorate.

A few questions remain open for further investigation. How can traditional surveys and social media digital traces be best combined to maximize their predictive ability? Can social media provide a reliable leading indicator to unemployment, and in general, economic surveys? How much reliable lead is it possible, if at all? As we have found, Twitter penetration and educational levels are found to be correlated with unemployment, but this levels are unlikely to change rapidly to describe or anticipate changes in the economy or unemployment. However, other indicators like daily activity, social interactions and geographical mobility are more connected with our daily activity and perhaps they have more predicting power to show and/or anticipate sudden changes in employment. The relationship between unemployment and individual and group behavior may help contextualize the multiple factors affecting the socioeconomic well-being of a region: while penetration, content, daily activity and mobility diversity seem to be highly correlated to unemployment in Spain, different weights for each group of traces might be expected in other countries [14]. Finally, digital traces could serve as an alternative (some times the only one available) to the lack of surveys in poor or remote areas [20, 28]. Another interesting avenue of research involves the use of social media to detect mismatches between the real (hidden, underground) economy and the officially reported [39].

Most importantly, the immediacy of social media may also allow governments to better measure and understand the effect of policies, social changes, natural or man-made disasters in the economical status of cities in almost real-time [18, 40]. Our results can also be framed within the emerging phenomenon of the shift of the digital divide [41], which shows that the current gap in online presence in developed countries is not due to digital access, but to the socio-economical situation of individuals. These new avenues for research provide great opportunities at the intersection of the economic, social, and computational sciences that originate from these new widespread inexpensive datasets.

## Supporting Information

S1 File This file contains Figures A-I, Tables A-F and Sections A-I. (PDF)
