# Supplementary material for: Social Media Fingerprints of Unemployment
Source: PLoS One. 2015 May 28;10(5):e0128692. doi: 10.1371/journal.pone.0128692 (PMC4447438; doi:10.1371/journal.pone.0128692)
Supplement: S1 File — (PDF) [file pone.0128692.s001.pdf]

S1 File
Supporting Information for
Social media fingerprints of unemployment

Alejandro Llorente, Manuel García-Herranz, Manuel Cebrián, and Esteban Moro

May 13, 2015

Contents

A. The datasets **2**

B. Twitter as mobility proxy **2**

C. Community structures in inter-city mobility graph **3**

D. Twitter demographics and unemployment rates **6**

E. Properties of Twitter variables **7**
E.1. Normalization and distributions 7
E.2. Correlation between variables 8

F. Misspellers detection **10**

G. Time window and unemployment **12**

H. Demographics does not explain unemployment **13**

I. Unemployment models for other geographical areas **14**

J. Relative importance of the variables **14**

A. The datasets

Twitter provides an extremely rich and publicly available data set of user interactions, information flows and, thanks to the geo location of tweets, user movements. Nevertheless, the representativeness of this geo-located Twitter as a global source of mobility data has still received sparse attention. In this sense, while [13] present a promising and extensive study regarding global country-to-country movements (mostly driven by tourism), within-country human flows (comprising not only internal tourism but also, in a greater extent than country-to-country travels, visiting and commuting) still need further investigation. Therefore, throughout this work we will compare our findings using geo-located Twitter with similar study using commuting surveys.

For the Twitter analysis, we consider 19.6 million geo-located Twitter messages (tweet(s)), collected through the public API provided by Twitter for the continental part of Spain and from 29th November 2012 to 10th April 2013. In this dataset we consider that there has been a trip from place l to place k if a user has tweeted in place l and place k consecutively. We only keep those transitions when the first tweet and the second one are dated in the same day. We filter the trips database to avoid unrealistic transitions and keep only trips with a geographical displacement larger than 1km. By this method, 1.38 million of trips from 167,376 different users are considered in our work.

From those trips we construct the mobility flow T_{ij} between municipalities, which measures the number of trips in our database in which the origin is within city i boundaries and destination lies within those of city j.

We also consider population and economical information about the municipalities from the Spanish Census (2011) [8] and unemployment figures from the Public Service of Employment (Servicio Público de Empleo Estatal, SEPE) [7]. In the latter case, registered unemployment (in number of persons) is given for each Spanish municipality by gender, age, and month. To get unemployment rates we divide register unemployment by the total workforce in the municipality, estimated as the number of people with age between 16 and 65 years.

All the collected data complies with the terms of service for the websites where they were downloaded.

B. Twitter as mobility proxy

Considering all of the available transitions in our database, one can compute the distance between origin and destination, the elapsed time of the transition and the number of trips per user among many other statistics. Using the method described in [26], the trip distance and the number of trips exhibit a clear Power-law distribution (KS statistics 0.05 and 0.06 with exponents -1.62 and -2.12 respectively) whereas for the elapsed times, the best option is to fit a exponentially-truncated Power-law distribution (KS statistic 0.046 with exponent -0.67). For all these parameters, focusing on the log-linear part of the distributions, self-similar behaviors arise when Twitter based mobility is analyzed (see Fig. A).

Twitter based inter-city flows can be well modelled by means of the The Gravity Law, which is one of the most extended methods to represent human mobility [1, 19] , with applications in many fields like urban planning [23], traffic engineering [4] or transportation problems [9]. Gravity Law

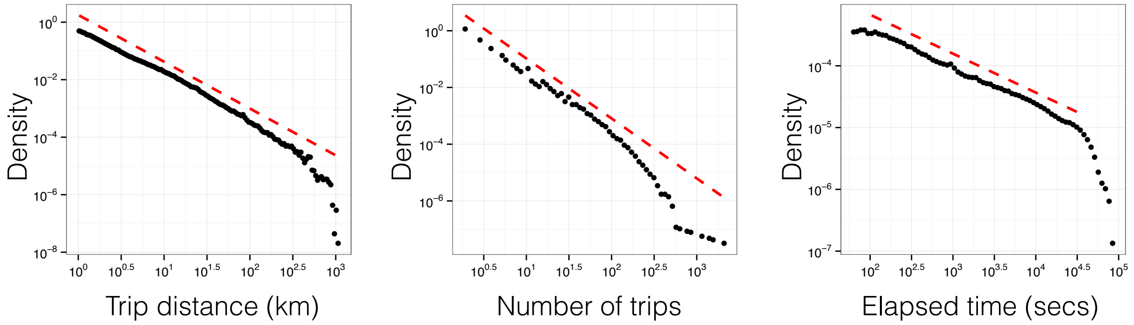

Fig A: Probability distributions for the different properties of daily trips in the Twitter dataset. Dashed lines corresponds to a power law fit with exponents of the log-linear bining -1.62, -2.12 and -0.67 respectively

is also the solution to the problem of maximizing the entropy of the particle distribution among all the possible trips using statistical mechanics techniques [22, 2]. Recently, it has also been used as a model for human mobility based on cell phone traces [20, 10, 21] and social media data at a global scale [13] and at the inter-city level [14].

The Gravity Model for human mobility assume that the flows between cities can be explained by the expression

$$T_{ij}^{grav} = \frac{P_i^{\alpha_1} P_j^{\alpha_2}}{d_{ij}^{\beta}} \tag{1}$$

where T_{ij}^{grav} is the flow, in terms of number of people, between cities i and j, d_{ij} is the geographical distance and P_i and P_j the population of every city respectively.

Given the data we can obtain the parameters of the model by Weighted Least Squares Minimization,

$$\alpha_1^*, \alpha_2^*, \beta^* = \underset{\alpha_1,\alpha_2,\beta}{\text{argmin}} \frac{1}{N} \sum_{i,j} w_{ij} \left(T_{ij} - T_{ij}^{grav}\right)^2 \tag{2}$$

where N is the total number of connections in the mobility graph and w_{ij} is a weight proportional to the number of observed transitions between i and j. In particular we find that taking $w_{ij} = T_{ij}^{1.3}$ gives the best performance in the model.

In our case, this model fits quite accurately the inter-city mobility based on Twitter GPS check-ins (see Table A). Even though we are considering T_{ij} not necessarily symmetric, the exponents of the populations are similar indicating that we are observing a similar flows in both directions between i and j.

C. Community structures in inter-city mobility graph

Typically, complex networks exhibit community structure, that is, there are subsets of nodes that are more densely connected among them comparing to the rest of the nodes. In mobility networks, whose nodes correspond to geographical areas, these communities are interpreted as zones with

Gravity Model		
Parameter	Description	Spain
α_1	Origin exponent	$0.477^{***}(0.002)$
α_2	Destination exponent	$0.478^{***}(0.002)$
β	Distance exponent	$1.05^{***}(0.0035)$
R^2	Goodness of fit	0.797
ϕ	Correlation between T_{ij} and T_{ij}^{gra}	0.826

Table A: Description of the parameters for the Gravity Law Model in geo-tagged social media data for Spain. $(***)$ means significance $p < 0.0001$.

high common activity and tend to be constrained by geographical and political barriers. We check whether this is also observed in our dataset by performing 6 state-of-art community detection algorithms: FastGreedy [5], Walktrap [16], Infomap [18], MultiLevel [3], Label Propagation [17] and Leading Eigenvector [15]. These six different algorithms exhibit different community structures in terms of number of communities, average size of community or modularity (see Table C). Members (municipalities) of the resulting communities are spatially connected except some few cases as Fig. C shows. We test the statistical robustness of the obtained communities by randomly removing a proportion p of the original links and performing the algorithms on this new graph G_p. We will consider that communities are robust when the communities given for the original network G and G_p are highly similar. In order to compare two arbitrary memberships to communities, we use the Normalized Mutual Information (NMI) method described in [6] which returns 0 when two memberships are totally different and 1 when we compare two equal memberships. We compute the NMI for each chosen algorithm performed on G and G_p, for p between 1% and 10%, concluding that obtained community structures are robust because they are not broken when some randomly chosen links are removed (see Table B).

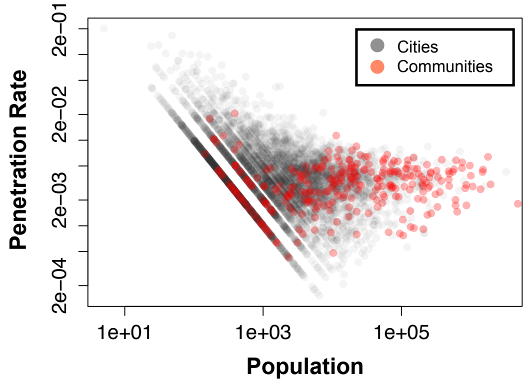

Fig B: Penetration rates for both cities and detected communities.

As other works have shown, mobility graph communities are usually interpreted in terms of geographical and political barriers and a natural question is whether the mobility based com-

NMI between G and G_p for different p										
Algorithm	$p = 0.01$	0.02	0.03	0.04	0.05	0.06	0.07	0.08	0.09	0.1
FG	0.995	0.992	0.989	0.983	0.981	0.977	0.983	0.969	0.980	0.959
WT	0.954	0.959	0.950	0.954	0.945	0.948	0.947	0.935	0.926	0.931
IM	0.988	0.981	0.980	0.981	0.978	0.974	0.975	0.970	0.969	0.966
ML	0.994	0.978	0.979	0.983	0.948	0.934	0.972	0.952	0.973	0.947
LP	0.906	0.908	0.911	0.915	0.895	0.907	0.907	0.893	0.905	0.904
LE	0.960	0.957	0.956	0.859	0.910	0.892	0.908	0.858	0.885	0.884

Table B: NMI measure comparing G and G_p.

munities are related to any of these barriers. In Spain, there are different territorial divisions for administration purposes. In this work, we consider two of them: provinces, defined in 1978 Constitution, are 50 different heterogeneous aggregations of municipalities; and counties (*comarca* in Spanish terminology) which are traditional aggregations of municipalities mainly based on Spanish orography (rivers, valleys, ridges, etc) and some of them are composed by municipalities of different provinces. We use again the NMI method to compare the communities structure given by the algorithms to the administrative limits. Except Leading Eigenvector algorithm, the rest of methods return communities that are quite related to provinces ($NMI \approx 0.7$) whereas for the county administration limits, higher variability is observed. In this last case, the algorithm providing more relationship with county limits is Infomap, $NMI \approx 0.83$. Therefore, Twitter based mobility summarizes the inter-city flows exhibiting that these flows are influenced by geographical and political barriers.

Communities Stats						
Algorithm	$\langle \lvert N_i \rvert \rangle_i$	$\max\{\lvert N_i \rvert\}$	$\lvert\{N_i\}\rvert$	Modularity	*NMI P*	*NMI C*
FG	309.1	1405	23	0.836	0.70	0.58
WT	8.91	453	798	0.791	0.73	0.76
IM	20.91	142	340	0.758	0.77	0.83
ML	284.36	1254	25	0.834	0.71	0.0.59
LP	21.157	750	336	0.730	0.73	0.75
LE	117.6	1509	61	0.748	0.56	0.49
Counties	22.4	112	318	0.57	0.81	1
Provinces	142.2	315	50	0.79	1	0.81

Table C: Statistics of the communities $\{N_i\}$ returned by the six algorithms. *NMI P* refers to the comparison between communities and provinces whereas *NMI C* considers counties instead of provinces.

As we can see, different algorithms also give different spatial resolutions. While FastGreedy, MultiLevel and LeadingEigenvalue yield to a small number of large partitions, we got a higher spatial resolution in the partitions obtained by WalkTrap, InfoMap and LabelPropagation. Since we want to study unemployment at a finer spatial scale than provinces, we consider only those

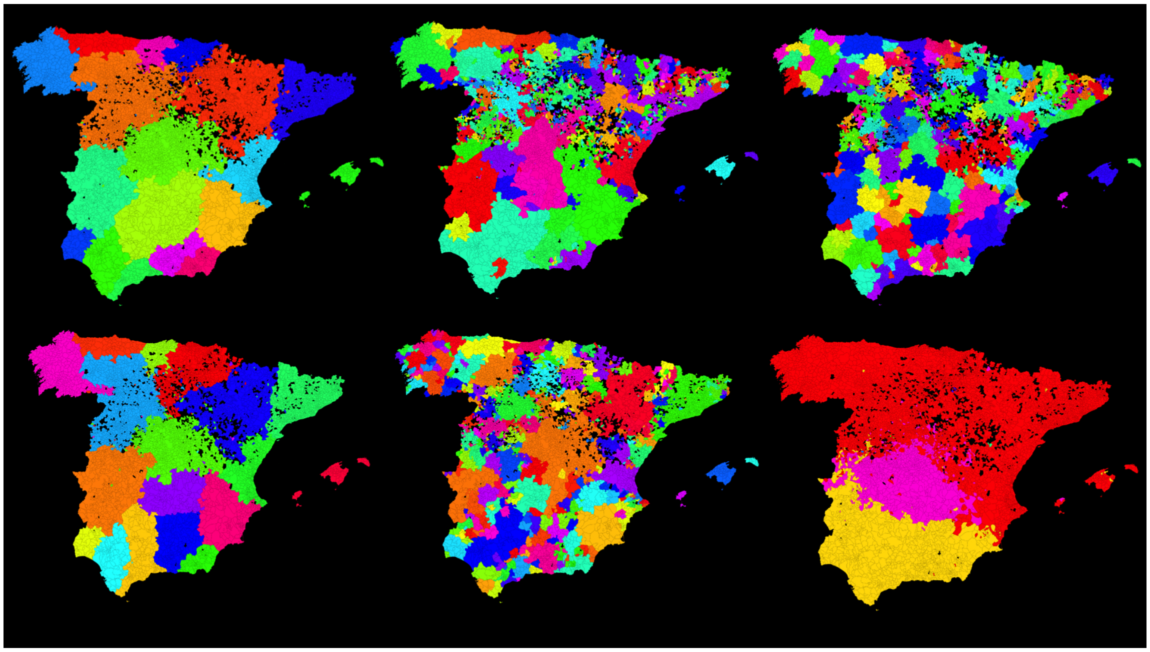

Fig C: From left to right and from top to bottom: Fastgreedy, Walktrap, Infomap, Multilevel, Label Propagation and Leading Eigenvector communities on Twitter based mobility transitions.

latter methods in our study. Note also that the counties partition has small modularity with the observed mobility graph and thus we have discarded it. Finally, in the main text we have used the partition obtained by InfoMap since, as explained before, they have more overlap with counties. However, as shown in Section I., our main results are similar for other partitions at different resolution levels. Specifically, LabelPropagation partition yields to very similar results as the InfoMap communities.

D. Twitter demographics and unemployment rates

Different age groups are not equally represented in Twitter. Recent surveys (2012) in Spain suggest that most (86%) of users in Twitter are 16 to 44 years old. Comparison of the percentage of users per age group with the total population within the same groups (see Fig. D) reveals that groups of ages above 35 years old are under-represented in Twitter. Thus our Twitter data will be more revealing when trying to describe unemployment in age groups below 44 years old. This is indeed what we find when we try to build a linear model for the rate unemployment in different age groups with the same Twitter variables: while unemployment rates for ages below 24 can be fitted to a linear model with $R^2 = 0.62$ we find that regression models for unemployment rates for ages between 25 and 44 have a $R^2 = 0.52$, while for ages above 44 we get only $R^2 = 0.26$. Table D summarizes the results for the regression models of unemployment rates in each age group, showing that our Twitter variables have more explanatory power for ages below 44. Finally, in

Fig. D we can see the performance of the model at different age groups and, once again, it is obvious the poor explanatory power of the Twitter variables for the unemployment rate in ages above 44 years old.

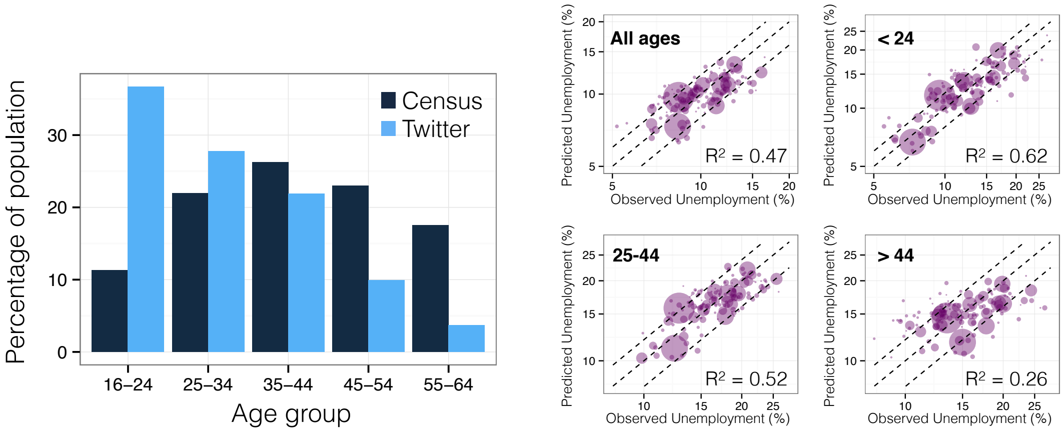

Fig D: Left: Percentage of population in each age group from the Spanish Census (dark bars) and surveys about users in Twitter (light bars). Right: performance of the linear models for each of the age groups.

E. Properties of Twitter variables

E.1. Normalization and distributions

Heterogeneity between the values of variables constructed from Twitter is large but moderate, as histograms in Fig. E show. We did not find any geographical area with anomalous values in any of the variables considered. Variables are normalized in different ways: both the penetration τ_i and misspellers rate ε_i are defined as the number of users or misspellers per 100.000 persons (population); activity variables ν_i are normalized as the percentage of tweets per time interval; finally, number of tweets that mention a specific term μ_i are also given per 100.000 tweets published in the geographical area.

Finally we have also considered potential bias in the entropy estimations due finite size effects of the sample, which could create spuriously high information values. To this end we have used the simple Miller-Madow correction to entropy estimation [24]. However, both the original and corrected estimations are highly correlated: for example, for the mobility entropies Pearson's correlation coefficient is 0.99 and MSE between both estimations is 0.09. Thus, there is no significant bias in our estimation of the entropies.

	All ages	< 24	$25-44$	> 44
(Intercept)	$0.11 ****$	0.10^{***}	0.20^{***}	0.20^{***}
	(0.02)	(0.03)	(0.03)	(0.035)
Penetration rate	3.23^{*}	8.57^{***}	6.28^{**}	2.40
	(1.41)	(2.22)	(2.17)	(2.77)
Geographical diversity	0.03	0.15^{***}	0.08^{*}	0.06
	(0.02)	(0.04)	(0.04)	(0.05)
Social diversity	-0.03^{*}	-0.03	-0.05^{*}	-0.06^{*}
	(0.01)	(0.02)	(0.02)	(0.03)
Morning activity	-0.69^{*}	-1.30^{**}	-1.53^{***}	-1.19^{*}
	(0.26)	(0.42)	(0.41)	(0.52)
Misspellers rate	11.56	31.51^{*}	15.46	23.60
	(8.13)	(12.78)	(12.48)	(15.94)
Employment mentions	-1.80	3.17	-9.94	2.71
	(6.27)	(9.86)	(9.64)	(12.3)
R^2	0.47	0.64	0.55	0.29
Adj. R^2	0.44	0.62	0.52	0.26

$^{***}p < 0.001$, $^{**}p < 0.01$, $^{*}p < 0.05$

Table D: Regression table for the different models in which unemployment for different age groups is fitted. The *All ages* model is the fit to the general rate of unemployment in each geographical area, while the other models are for the rates of unemployment in groups of less than 24 years, between 25 and 44 years and above 44 years.

E.2. Correlation between variables

Variables are constructed to reflect the behavior of areas in the different dimensions of Twitter penetration, social or geographical diversity, activity through the day and content. Correlation between variables does indeed show that variables within each dimensions hold strong correlations between them. As we can see in Fig. F social and geographical diversities are highly correlated between them, an expected fact given the *gravity law* accurate description of flows of people between geographical areas, but also the amount of communication between them. Same behavior is found for the group of variables in the activity group, while content variables are less correlated. Finally we find that both the penetration rate τ_i and fraction of misspellers ε_i have a strong correlation with most of the variables.

High correlation between variables might lead to collinearity effects [25] in the linear regression models, that is, some variables with predictive variable might have non-significant weights because they explain the same part of the variance. For instance, in Table E misspellers rate has a very strong predictive value but its p-value is too high to consider it significant. To test this hypothesis, we perform a principal component analysis (PCA) on the independent variables of the regression. Fig. F exhibits the loadings of the different variables for the considered variables. The block structure showed in F results in similar directions of the variables in the first componentes of the PCA. We observe some groups of variables: on the one hand, geographical and social diver-

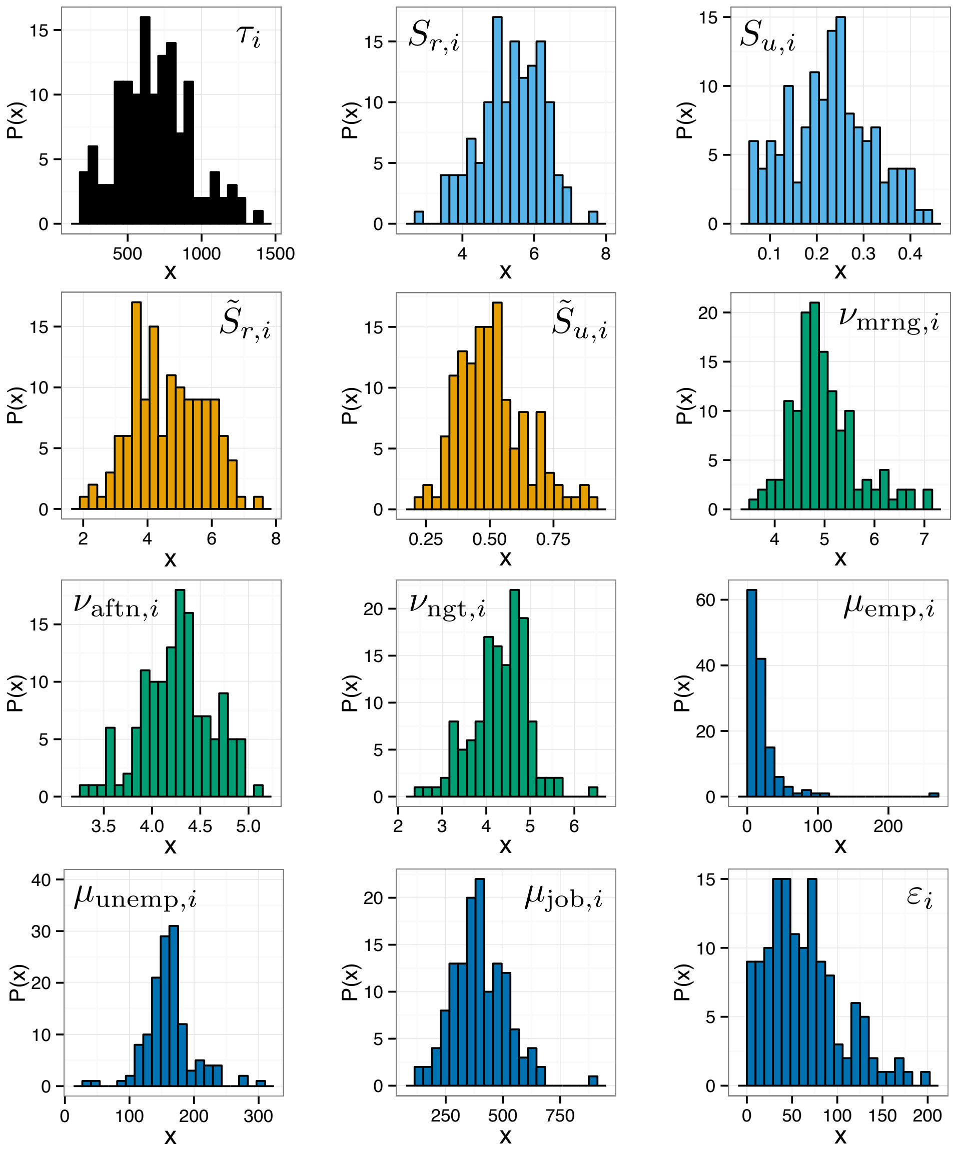

Fig E: Frequency plots for each variable constructed from Twitter.

sity seem to explain large part of the variance; on the other hand, we find a perpendicular group of variables formed by temporal activity; finally, penetration rate and misspellers fraction seem to represent a different independent direction of data, with high collinearity between them. This might explain the low statistical significance in the models of section I.. In any case, the structure of the correlation matrix and the PCA results show that there is indeed information in all groups

of variables and thus we have take a variable in each of them for our regression models.

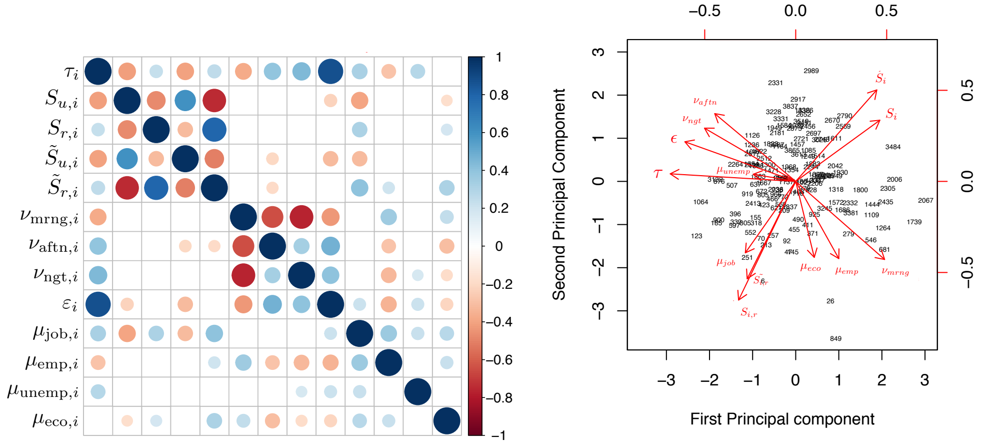

Fig F: Left: Correlation matrix between the variables constructed from Twitter. Each entry in the matrix is depicted as a circle whose size is proportional to the correlation between variables and the sign is blue/red for positive/negative correlations. Blank entries correspond to statistically insignificant correlations with %95 confidence. Right: Variables projection on the first two principal components given by PCA. We observe different groups of variables and collinearity between some of them.

F. Misspellers detection

In this work we will consider only tweets in Spanish, that is, since in Spain several languages live at the same time, depending on the part of the country, the first step is to reduce our Twitter dataset to those tweets that are written in Spanish. This task is carried out using the n-gram based text categorization R library *textcat* [11]. Then, in order to decide whether a tweet has a misspelling or not, we need to establish some patterns to select from our set of tweets. Since we want to be sure that a detected mistake corresponds to a real misspeller, we will not consider the following cases:

- Lack of written accents. People tend to avoid writing accents when talking in a colloquial way.
- Mistakes derived from removing *unnecessary* letters. The most common cases are removing a *h* at the beginning of a word (in Spanish the letter *h* is not pronounced), or replacing the letters *qu* by *k*. We understand that these mistakes can be motivated for the limitation of length in tweets, and not for a real misspelling.
- In the same line, we neglect mistakes produced by removing letters in the middle of a word, whose pronunciation can be deduced without them.

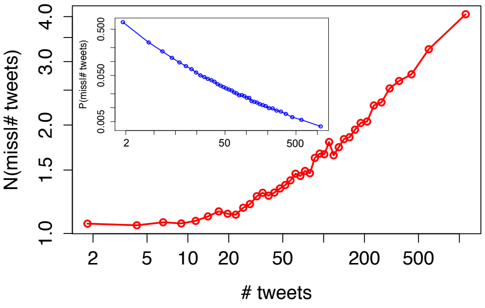

Fig G: Number (red) and probability (blue) of observed misspellings given the number of tweets.

- We do not consider either mistakes related to features of specific areas in Spain. For example, in the south the pronunciation of *ce* and *se* is the same, what produces a big amount of mistakes when writing. However, since we want to extract objective and equitable conclusion over the whole Spanish geography, we neglect those misspellings that only appear in a specific area.

Likewise, we will consider as real misspellings the following mistakes:

- Adding letters. For example, writing a *h* at the beginning of a word that starts with a vowel.
- Changing the special cases *mp*, *mb* by the wrong writings *np*, *nb*.
- Mixing up *b* with *v*, *g* with *j*, *ll* with *y*, and *ex* with *es*. These are typical mistakes in Spanish, because they have the same, or a very close, pronunciation.
- Confusing the verb *haber* with the periphrasis *a ver*.
- Separating a word into two ones, for instance, writing the word *conmigo* as *con migo*.

This way, our list of mispellings is composed of 617 common mistakes in Spanish, that cannot be attributed to the special features of Twitter or a specific region of Spain. Thus, one can expect that this selection provides an accurate and equitable method of detecting misspellers. Under these conditions, the number of users who wrote at least one misspelled word is 27055 (5.6% over the whole population).

We analyze whether misspellers have different Twitter usage behavior from that people who do not make serious mistakes when publishing a tweet. Comparing the average number of tweets, it can be observed that misspellers tend to publish a larger number of tweets than those who did not made mistakes (144.71 against 23.72). This also emerges when the mean number of misspelling given the total number of tweets is considered. For users with less than approximately 30 published tweets in the observation period, the number of misspellings is almost zero whereas for users who publish more often, the mean number of misspellings scales sub-linearly with the number of tweets ($exponent \approx 0.33$).

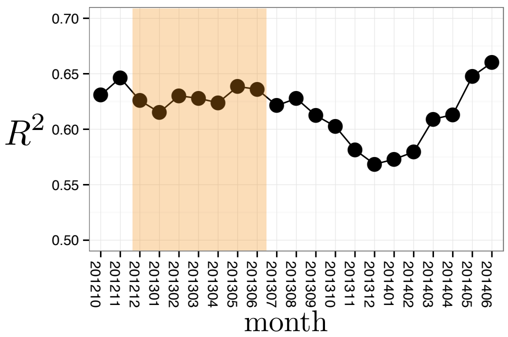

Fig H: Explanatory power of the linear regression model when fitted against the unemployment data for different months. Gray (orange) area correspond to the time window in which Twitter data is collected and variables are constructed.

Since we have observed a segmentation of Twitter population based on how accurate they write, we consider the misspeller rate as a proxy of the educational level of the cities. Large number of previous works in the literature have revealed the relationship between the economical status and the educational level of geographical areas and therefore it is natural to ask whether the observed misspellers rate is related to economy driven by the unemployment rate. To test this hypothesis, we consider cities populated with more than 5000 inhabitants to avoid subsampled cases. We find a strong positive correlation between the probability of finding a misspeller in a city and the unemployment rate (0.372, 0.491).

G. Time window and unemployment

In the definition of the variables we have aggregated the Twitter activity within a 7 months time window (from December 2012 to June 2013). Since unemployment has a significant variation along time, we investigate here what is the correlation and explanatory power of the Twitter variables for the values of unemployment determined at different months through the same time window in which Twitter data was collected. Or if the variables collected in that time window are more correlated with past or future values of unemployment. Fig. H shows the explanatory value of the model when the linear regression is done for values of unemployment of different months before, during and after the Twitter data time window. Although there is a small seasonal effect along the year, we see that the explanatory power remains around $R^2 = 0.6$, which suggest that our Twitter linear model retains its explanatory power even though unemployment changes considerably throughout the year. It is interesting to note that R^2 decays a little bit during the summer which means that our variables are less correlated with summer unemployment. Finally, unemployment used in the main article is from June 2013, i.e. the last month in the time window used to collect the data.

	All variables	Youth model	Twitter model (I)	Twitter model (II)
(Intercept)	0.06	−0.02	0.10***	0.09***
	(0.03)	(0.03)	(0.03)	(0.027)
Young pop. rate	0.66*	2.20***		
	(0.30)	(0.35)		
Penetration rate	8.20***		8.57***	8.62***
	(2.25)		(2.22)	(2.21)
Geographical diversity	0.14***		0.15***	0.12***
	(0.04)		(0.04)	(0.03)
Social diversity	−0.02		−0.03	
	(0.02)		(0.02)	
Morning activity	−1.42***		−1.30**	−1.28**
	(0.41)		(0.42)	(0.41)
Misspellers rate	23.95		31.51*	32.28*
	(13.09)		(12.78)	(12.71)
Employment mentions	0.34		3.17	
	(9.81)		(9.86)	
R^2	0.65	0.24	0.64	0.63
Adj. R^2	0.63	0.24	0.62	0.62

$^{***}p < 0.001$, $^{**}p < 0.01$, $^{*}p < 0.05$

Table E: Regression table for the different statistical models. The *All variables* model includes both Twitter and rate of young population variables. *Twitter model (I)* includes only the variables described in the main article, while *Twitter model (II)* only includes those variables which are significant $p < 0.05$ in *Twitter model (I)*.

H. Demographics does not explain unemployment

Since unemployment rates are very large for the group of young people, a natural question is whether only demographic variables could explain the heterogeneity of young unemployment rates found in the geographical areas. To test this end we have built four linear models: the first one (named *Youth model* in Table E) is composed by the rate of young population as the only explaining variable; the second ones are built based on only the Twitter variables considered in the main text (named *Twitter model (I)*) or just with those whose regression coefficients are statistically significant (*Twitter model (II)*); the third one is fitted with all the variables (named *All variables* model in Table E). In Table E we show the summary of the regression for each model. Focusing on the explained variance by the model in terms of R^2, it can be checked that considering all Twitter variables is three times more explanatory than considering only the young people proportion. On the other hand, the comparison of R^2 for the *Twitter model* with the one for *All variables* and *Youth model* shows that the rate of young population does not provide a significant explanatory power. This semi-partial analysis shows that our Twitter variables retain a high explanatory power when the effect of young population rate is controlled.

I. Unemployment models for other geographical areas

While municipalities are very heterogeneous demographically, other administrative areas exist in Spain at large scales that could be used for our model of unemployment. As mentioned in section C., the smallest administrative division of Spain we have considered is that of the 8200 *municipalities*. At larger scales we have the 326 *counties* (*comarcas* in spanish) which are aggregations of municipalities. Finally, the largest geographical scale we considered is defined by 50 provinces (*provincias* in Spanish). In this section we compare the performance of our Twitter model for unemployment for the variables defined in those administrative areas and relate it to the geographical communities detected and used in the main paper (see section C.). Not all the areas at different administrative divisions are considered in the model. To minimize the effect of areas in which the number of geo-tagged tweets is very small, we only consider the 1738 municipalities which have a Twitter population $\pi > 10$. Similarly, we only consider the 198 counties with $\pi > 100$. As we can see in Table F the model has a large explanatory power for areas equal or bigger than counties. As expected R^2 increases as the number of areas in the model is smaller, but the description level of the model is very low for provinces, for example. The best performance (high R^2 and high geographical description level) is attained at the level of the detected communities. Other partitions obtained with a different community-finding algorithm yield to similar results, as shown in Table F for LabelPropagation.

J. Relative importance of the variables

To asses the relative importance of the variables in the unemployment model we have used several methods. They all give qualitatively the same results, with some variations for the statistically insignificant variables. Specifically, we have use

1. *(weight)*: Relative weight of the absolute values of the coefficients obtained in the linear regression when variables are scaled to have mean zero and variance one.
2. *(lmg)*: averaging over orderings, proposed by Lindeman, Merenda and Gold
3. *(pmvd)*: The PMVD metric introduced by Feldman which an average over orderings as well, but with data-dependent weights
4. *(first)*: The univariate R^2-values from regression models with one variable only.

All these metrics are obtained using the *relaimpo* R package [12]. The results for the young unemployment model are shown in Fig. I where we can see that different methods yield to similar relative importance of the variables, excepting perhaps for the diversity of mobility flows, a variable with a non-significant weight in the regression model.

References

[1] B Ashtakala. Generalized power model for trip distribution. *Transportation Research Part B: Methodological*, 21(1):59–67, 1987.

	Communities (IM)	Communities (LP)	Municipalities	Counties	Provinces
(Intercept)	0.10***	0.05	0.16***	0.11***	0.11*
	(0.03)	(0.03)	(0.01)	(0.03)	(0.05)
Penetration rate	8.57***	11.44***	4.01***	9.12***	10.47***
	(2.22)	(2.31)	(0.59)	(1.81)	(1.97)
Geographical diversity	0.15***	0.14***	0.02	0.12***	0.08
	(0.04)	(0.04)	(0.01)	(0.03)	(0.07)
Social diversity	−0.03	−0.01	−0.01	−0.01	−0.03
	(0.02)	(0.02)	(0.01)	(0.02)	0.07
Morning activity	−1.30**	−0.73	−1.16***	−1.49***	−1.03
	(0.42)	(0.44)	(0.14)	(0.39)	(0.88)
Misspellers rate	31.51*	17.47	14.40***	14.09	
	(12.78)	(12.13)	(2.51)	(10.02)	
Employment mentions	3.17	4.81	−0.71	2.41	−3.17
	(9.86)	(11.42)	(0.89)	(8.86)	(12.29)
Number of points	128	121	1738	198	50
R^2	0.64	0.63	0.22	0.55	0.65
Adj. R^2	0.62	0.61	0.21	0.54	0.61

$^{***}p < 0.001$, $^{**}p < 0.01$, $^{*}p < 0.05$

Table F: Regression table for the unemployment linear regression model in different levels of geographical areas. The first two columns show the result of the model for the communities detected in the Infomap (main text) and LabelPropagation algorithms. In the *Provinces* model, the misspellers rate has been removed from the model due to the large collinearity with the penetration rate.

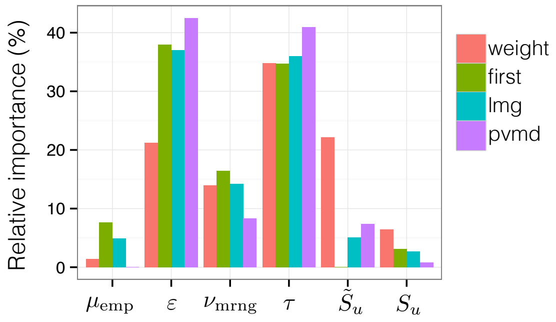

Fig I: Relative importance of the variables (in percentage) in the unemployment model for different ways to calculate it.

[2] Michel Bierlaire. Mathematical models for transportation demand analysis. *Transportation research. Part A, Policy and practice*, 31(1):86–86, 1997.

[3] Vincent D Blondel, Jean-Loup Guillaume, Renaud Lambiotte, and Etienne Lefebvre. Fast unfolding of communities in large networks. *Journal of Statistical Mechanics: Theory and Exper-*

iment, 2008(10):P10008, 2008.

[4] Harry J Casey Jr. The law of retail gravitation applied to traffic engineering. *Traffic Quarterly*, 9(3), 1955.

[5] Aaron Clauset, Mark EJ Newman, and Cristopher Moore. Finding community structure in very large networks. *Physical review E*, 70(6):066111, 2004.

[6] Leon Danon, Albert Diaz-Guilera, Jordi Duch, and Alex Arenas. Comparing community structure identification. *Journal of Statistical Mechanics: Theory and Experiment*, 2005(09):P09008, 2005.

[7] Servicio Público de Empleo Estatal (SEPE). Spanish registered unemployment. `http://www.sepe.es/contenidos/que_es_el_sepe/estadisticas/index.htm`.

[8] Instituto Nacional de Estadística. Spanish 2011 census. `http://www.ine.es/censos2011_datos/cen11_datos_inicio.html`.

[9] Suzanne P Evans. A relationship between the gravity model for trip distribution and the transportation problem in linear programming. *Transportation Research*, 7(1):39–61, 1973.

[10] Paul Expert, Tim S Evans, Vincent D Blondel, and Renaud Lambiotte. Uncovering space-independent communities in spatial networks. *Proceedings of the National Academy of Sciences*, 108(19):7663–7668, 2011.

[11] Ingo Feinerer, Christian Buchta, Wilhelm Geiger, Johannes Rauch, Patrick Mair, and Kurt Hornik. The textcat package for n-gram based text categorization in r. *Journal of Statistical Software*, 52(6):1–17, 2013.

[12] Ulrike Grömping. Relative importance for linear regression in r: the package relaimpo. *Journal of statistical software*, 17(1):1–27, 2006.

[13] Bartosz Hawelka, Izabela Sitko, Euro Beinat, Stanislav Sobolevsky, Pavlos Kazakopoulos, and Carlo Ratti. Geo-located twitter as the proxy for global mobility patterns. *arXiv preprint arXiv:1311.0680*, 2013.

[14] Yu Liu, Zhengwei Sui, Chaogui Kang, and Yong Gao. Uncovering patterns of inter-urban trips and spatial interactions from check-in data. *arXiv preprint arXiv:1310.0282*, 2013.

[15] Mark EJ Newman. Finding community structure in networks using the eigenvectors of matrices. *Physical review E*, 74(3):036104, 2006.

[16] Pascal Pons and Matthieu Latapy. Computing communities in large networks using random walks. In *Computer and Information Sciences-ISCIS 2005*, pages 284–293. Springer, 2005.

[17] Usha Nandini Raghavan, Réka Albert, and Soundar Kumara. Near linear time algorithm to detect community structures in large-scale networks. *Physical Review E*, 76(3):036106, 2007.

[18] Martin Rosvall and Carl T Bergstrom. Maps of random walks on complex networks reveal community structure. *Proceedings of the National Academy of Sciences*, 105(4):1118–1123, 2008.

[19] Morton Schneider. Gravity models and trip distribution theory. *Papers in Regional Science*, 5(1):51–56, 1959.

[20] Filippo Simini, Marta C González, Amos Maritan, and Albert-László Barabási. A universal model for mobility and migration patterns. *Nature*, 484(7392):96–100, 2012.

[21] Chaoming Song, Tal Koren, Pu Wang, and Albert-László Barabási. Modelling the scaling properties of human mobility. *Nature Physics*, 6(10):818–823, 2010.

[22] Alan Geoffrey Wilson. *Entropy in urban and regional modelling*. Pion Ltd, 1970.

[23] Alan Geoffrey Wilson. Urban and regional models in geography and planning. 1974.

[24] G. Miller. Note on the bias of information estimates. *Info. Theory Psychol. Prob. Methods*:95-100.

[25] Svante Wold, Arnold Ruhe, Herman Wold, and WJ Dunn, III. The collinearity problem in linear regression. the partial least squares (pls) approach to generalized inverses. *SIAM Journal on Scientific and Statistical Computing*, 5(3):735–743, 1984.

[26] Jeff Alstott, Ed Bullmore and Dietmar Plenz powerlaw: a Python package for analysis of heavy-tailed distributions *PLoS One*, 9(4): e95816.
